# Finding sense of coherence in the menopause transition - A qualitative interview study with menopausal women in Norway

**DOI:** 10.1177/17455057261459816

**Published:** 2026-06-10

**Authors:** Marianne Natvik, Mette Brekke, Siri Vangen, Holgeir Skjeie

**Affiliations:** 1 Department of General Practice, Faculty of Medicine, 213459 University of Oslo, Oslo, Norway; 2 Norwegian Research Centre for Women’s Health, Oslo University Hospital, and Clinical Medicine, 155272 University of Oslo, Oslo, Norway; 3 General Practice Research Unit, 6305 University of Oslo, Oslo, Norway

**Keywords:** menopause, perimenopause, general practice, health education, women’s health, sense of coherence

## Abstract

**Background:**

Women express lack of knowledge about the menopause transition, finding it hard to understand and navigate symptoms. This period may negatively influence quality of life and level of functioning.

**Objectives:**

To explore menopausal women’s experiences, perspectives and coping strategies to comprehend, manage and find meaning in the menopausal transition.

**Design:**

Individual interviews of women with different backgrounds and menopausal experiences, recruited by hairdressers in Norway.

**Methods:**

Semi-structured in-depth interviews were conducted with 11 women in 2024. Data were analysed using reflexive thematic analysis. The theory of Salutogenesis and Sense of Coherence developed by Antonovsky, was used as guiding theoretical lens.

**Results:**

Three main themes were developed, incorporating the intercorrelated aspects of *Sense of Coherence*: Comprehensibility, Manageability and Meaningfulness. 1. *A transition from the unknown to normalisation –* without knowledge, understanding menopausal changes was challenged, but normalisation of menopause gave comprehension. 2. *From distress and silence to empowerment –* manageability increased with comprehension, discussions with other women and knowledge dissemination, but was challenged by inadequate health care. 3. *Finding meaning in the change menopause brings –* meaningfulness motivated to seek understanding and coping strategies, and gave acceptance and value to this transition, although often accompanied by an undesirable sense of aging.

**Conclusions:**

This study shows the importance of enhancing knowledge among women, the public and general practitioners to understand and manage menopause, and to improve menopausal care and women’s quality of life.

## Plain language summary

The menopause transition may give various symptoms that challenge women’s quality of life and functioning. Without knowledge about menopause, it can be hard to understand what is happening as symptoms may be unknown to be related to this life phase. This study used in-depth individual interviews with 11 menopausal women about their strategies to cope during this transition. The women expressed frustration over their inability to comprehend the symptoms, as they did not associate them with menopause until the onset of the well-known hot flashes. At the same time, they appreciated the increased discourse in society about the menopause transition that normalised this life phase and gave them a feeling of not being alone. Better understanding of the symptoms made it possible to try to manage the changes, through discussions with others and by seeking help. Several women experienced a feeling of not getting the help they expected from their general practitioner (GP) and were also surprised by the GP’s relative lack of knowledge. Still, they kept looking for answers and strategies to understanding and to find solutions for better care. Many found meaning in the changes the menopause transition gave, through new opportunities and an awareness that made them look at their lives and their health strategies, although they at the same time often acknowledged an undesirable sense of aging. This study shows the importance of providing knowledge to women, the society and doctors about the menopause transition to improve understanding and management and finally enhance women’s quality of life.

## Introduction

The menopause transition is known to influence women’s quality of life.^1,2^ Hot flashes, the hallmark of the menopause transition, is experienced by 80%,^3^ with about one third experiencing daily bothersome symptoms.^4^ The symptom profile also includes bleeding disturbances, mood and cognitive changes, sleep disruption, urogenital symptoms, and loss of libido.^2^ Fatigue, headache, anxiety, and musculoskeletal pain may also occur.^2,5^ The symptom burden varies between women. For some symptoms last several years, starting years before her last bleeding,^6^ while others experience few or no symptoms,^7^ and not all symptoms differentiate for the menopause transition.^6^ The experience of the life phase is related to the symptoms, but also to culture, ethnicity and race, general health and life adversities, knowledge and expectations, and the help received if consulting a doctor.^8–11^

Women* in many parts of the world express lack of sufficient knowledge about menopause**.^12–14^ Knowledge helps to recognise and manage symptoms, supports the experience, and may positively change women’s attitude towards menopause.^9,10,12,15,16^ Information sources about menopause are many, including, friends, family, colleagues, social media, general practitioners (GPs), gynaecologists, and the internet.^13,17^ However, women often express that the information available can be conflicting and inadequate.^9,10,12^

Studies highlight the need for empathetic, consistent medical advice to help women understand their experiences and make informed choices about self-care and treatment.^9,10,18^ Information provided by medical doctors can vary. Some focus mainly on menopausal hormone therapy (MHT) and medication, giving less attention to lifestyle and alternative treatments, resulting in women not being presented with all treatment options.^9,10,19^ Others might be reluctant to prescribe MHT due to viewing menopause as medicalised^20^ or to the perceived risk.^21–25^ With improved awareness, the prescription of MHT has increased over the past 10 years,^26,27^ but with inequities between women and between doctors.^23,25^

In Norway every inhabitant is assigned to a GP. There are about 5700 GPs, with an average of 944 patients each.^28^ The GP is first contact point for most medical issues and serves as gate keeper to publicly employed specialists, as a gynaecologist. Alternatively, patients may seek help directly from a private clinic with shorter waiting list but at a higher cost.

Salutogenesis and Antonovsky’s concept Sense of Coherence^29,30^ may in the menopausal context serve as a point of reference. Salutogenesis focuses on factors that promote health and well-being in contrast to pathogenesis that focuses on what causes or increases risk of disease.^29^ Salutogenesis aims to identify and enhance resources to cope despite stress and maintain health and quality of life. Sense of Coherence has three elements^30^: Comprehensibility: The belief that life events are structured, predictable, and understandable. Manageability: The belief that one has resources to cope with life’s challenges and demands. Meaningfulness: The belief that life has purpose and significance, which motivates individuals to engage and strive despite difficulties. Menopause might challenge all components.

The aim of this study was to explore strategies women use in menopause, using salutogenesis as a guiding theoretical lens. Our research question was: What coping strategies, information needs, and views on the GP’s role do women in menopause make use of to help themselves?

## Method

### Study design and recruitment

In this qualitative individual interview study using reflexive thematic analysis, participants were recruited through convenience sampling by hairdressers in Norway in both central and rural places. We acknowledge that hairdressers get to know their costumers and found this recruiting method feasible. The women included had to speak Norwegian and self-identify as being in the menopause transition with symptoms, following a natural transition. Exclusion criteria were menopause (last menstrual period) before age 45, treatment-induced menopause (e.g. due to breast cancer or gynaecological disease, including surgical menopause), and women without menopausal symptoms. We have no recording of non-participation as the hairdressers expressed that they recruited everyone they asked. All participants gave informed written consent. No compensation was given for participation.

The Regional Committee for Medical and Health Research Ethics in Norway has approved this study (ref. no. REK 716838).

### Sample

First author interviewed 11 women, age 49 - 56 years, between May and August 2024. Participant characteristics are presented in Table 1. Names given in the result section are pseudonyms.

**Table 1.** Demographic characteristics of participants (N=11).

Variables	n	Median (range)
**Age (years)**	​	54 (49-56)
**Rural/urban**		
Rural	3	​
Smaller city	4	
Big city	4	
**Marital status**		
Lives with partner	8	​
Lives alone	3	
**Children**		
0	1	​
1	1	
2	8	
4	1	
**Education**		
High school (10-12 years)	4	​
Higher education (>12 years)	7	
**Work situation**		
Full time work	8	​
Part time work due to sick leave	1	
Work disability	2	
**GP sex**		
Male	7	​
Female	3	
Do not know due to changes	1	

### Dataset generation

The interviews were done by MN using a flexible semi-structured interview guide (Appendix 1), developed and piloted by the research group. The participants chose the interview location, either their homes, workplaces, or MN’s research department. The interviews lasted 66 minutes median (61-77). They were recorded and saved on a digital platform within the safe area of the University of Oslo. MN transcribed all interviews verbatim within this platform and anonymised all data. The transcripts and findings were not shared with the participants. Sample size was guided by information power.^31^ After 11 interviews the research group evaluated the data material to be strong enough for the aim of this study.

### Data analysis

Reflexive thematic analysis was used to construct themes based on the research question.^32^ This process involves familiarising with the data, generating codes of valuable parts of the dataset and interpreting patterns to group shared meanings into themes. It is an iterative process, involving a continuous reviewing of the data, codes and themes.^33^ The analysis was initially explorative and inductive, with salutogenesis in mind, but without engaging actively in the theory. When all interviews were coded, the further analysis engaged in salutogenesis and sense of coherence in a more deductive way to broaden the analysis.

After three interviews, the research group met and conducted a preliminary thematic discussion as part of an interim analysis, with emphasis on the research question and the interview guide. Prior to this, all authors reviewed the interviews to familiarise themselves with the data. Last author (HS) also listened to the recordings.

Then MN and HS met to discuss and code the first interview together. The result of this initial coding-meeting was used as an open guide for further coding. The guide was not used as a framework but gave a start for a common understanding on how to approach the material in a reflexive manner. It was continuously shaped in the subsequent coding process, for which MN had the main responsibility, but in close discussions with the research group when needed. Coding was done using software NVivo (version 15).

After all interviews were coded, the research group met again. The authors read all interviews, and HS listened to all recordings. With an open mind we discussed what the data set contained and what stories the participants shared. We started to create a common understanding of code groups and eventually develop themes with subthemes. In this meeting we engaged in salutogenesis as a theory. The preliminary grouping of codes became more apparent through the theoretical guiding lens of salutogenesis. Sense of coherence was used in a deductive, but still reflexive, way in the development of themes and subthemes.

MN then revisited the data set with a stronger emphasis on a salutogenic perspective with an ongoing dialogue with the research group to further develop themes and subthemes. This involved a continuous evaluating and reevaluating of the dataset and engagement with the theory, rereading of the interviews and revision of codes and themes.

The final report was written by MN and then reviewed by MB, SV and HS. We did not share the results with the participants for validation. We have used the Consolidated criteria for reporting qualitative research (COREQ)^34^ as a guide throughout this project (Appendix 2).

### Reflexivity

Our preconceptions, ability to engage reflexively with the data and scientific position are part of the analysis process.^35,36^ Reflexivity has been part of the journey in this project. Potential biases, preconceptions, assumptions, and motivations have been acknowledged and reflected upon in meetings, including a log, fieldnotes after each interview and meeting minutes.

On a personal level, first author (MN) is a 48-year-old perimenopausal woman and a GP with special interest in women’s health. She gives menopause lectures to colleagues, medical students, and in public arenas, meets menopausal patients in a clinic and is author of a book about menopause. Her work and engagement shape her perspectives on midlife women and her research field. In the research group we are all medical doctors, (SV gynaecologist, the remaining GPs). HS, SV and MB are experienced researchers. HS is male, the remaining female. These experiences and characteristics have influenced the research process, our discussions and reflections.

On an interpersonal level a reflexive dimension is the power balance in the interview setting, a doctor doing the interview may give response bias (that the participants tell what the interviewer wants to hear). MN aimed to remain open to all viewpoints, and participants were invited at the end of each interview to raise any additional topics. MN informed the participants about her background and engagement in the research project.

Our basic epistemological position is critical realism.^37^ As medical doctors we believe there is an objective reality independent of our perceptions, while also recognising that our understanding of this reality is socially constructed and differs between individuals, influenced by factors like culture, language and context. We have explored the participants life experiences and are reporting on their shared experiences and events. As critical realists we have analysed the interviews within the frame of the reality we believe exists while also acknowledging structures that influence what we observe.

## Results

### Main themes and subthemes

**Figure 1.**
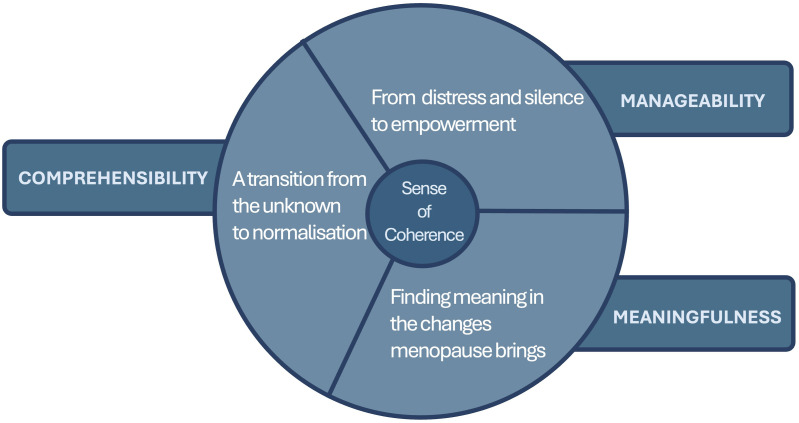
Three main themes in the article finding sense of coherence in the menopause transition.

We developed the themes with salutogenesis as a guiding theoretical lens in a reflexive process. The main themes were placed in a menopause specific three-component structure of Sense of Coherence: comprehensibility, manageability, and meaningfulness. The components intertwine and influence each other, and the themes interact, and are not strictly internally homogenous and externally heterogenous as often desired.^33^ But this also reflects the complexity of finding sense of coherence in the menopause transition. The resulting three themes and ten subthemes are presented in Table 2 and Figure 1.

**Table 2.** Themes, subthemes, and representative quotations.

Theme	Subtheme	Quote	SOC^*^ component
**1. A transition from the unknown to normalisation**			
**1.1**	**The unknown takes over until the hot flashes bring clarity**	“The challenges I’ve had, what can I pin them on? That’s the problem, you know, when we are forgetful, right? And I’ve actually asked if I could get a dementia test, you know. Then it might turn out later that it’s stress. Is it menopause. Is it… I don’t know.” Louise	**Increasing comprehensibility**
		“You might not understand. It sneaks up on you, you know. But like, not all the symptoms are exactly... I was waiting for the hot flushes.” Anne	
**1.2**	**Normalisation – “It’s not only me”**	“Yes, because I want to know about different experiences. ‘What I feel, do you feel the same? Is it like this for you? Do you experience it that way?’ There is something about not being alone, that there are others who experience the same. It is normal to experience this low mood. It is normal to feel this way. That is what I want to confirm.” Sara	
**2. From distress and silence to empowerment**			
**2.1**	**Why has no one told me how to face this phase of life?**	“Knowledge and security, really, go hand in hand for me.” Anne	**Managing menopause**
		“I also have a responsibility myself to get information. The GP is very busy and can’t inform everyone about this.” Sara	
**2.2**	**Let’s talk about it, but not before I am ready, and not about all symptoms**	“It can also be a case of ‘no, that’s not me,’ right? So it’s really about both parts, I think. It’s not just that; I believe there are many general practitioners who can contribute. But there needs to be a recipient who is willing to engage.” Ellen	
		«I can see that younger friends are like ‘talk to my hand.’ They don’t want to hear about menopause at all. » – laughs «And then I say, ‘I wish it had been a bit more prepared, are you sure…?' And these are people who work in healthcare and are openminded. No, it’s not relevant for them; they find it incredibly boring when the rest of us talk about menopause. Because they’re not there themselves. » Anne	
**2.3**	**My doctor is important but has no clue – how is that possible?!!**	“I think, this affects half the population, so I don’t understand why this isn’t straightforward at a general practitioner’s office.” Anne	
**2.4**	**I want my doctor to be curious**	“Instead of me sitting around trying to search and figure everything out while being on sick leave for months, I believe that a little curiosity can prevent that.” Anne	
**2.5**	**I can choose to influence by putting myself on the agenda**	“I actually feel that now it’s even more important to take care of myself and not to fight against it. It is a part of the cycle, and now I am getting help to ease the discomfort. But in a way, it’s about accepting what is happening with my body, not resisting it. Instead, I try to do everything I can to alleviate the discomfort and make things better.” Elisabeth	
**3. Finding meaning in the changes menopause brings**			
**3.1**	**Menopausal symptoms as a sign of an unwanted aging process**	“There have been physical changes that I dislike. I dislike that my skin changes, that the smells change, and that I look different. It’s all part of aging, right?” Dora	**Finding meaningfulness**
**3.2**	**This is important, I need to understand**	“I want to support the idea of approaching this with curiosity and a bit like, ‘Okay, now we’re stepping into a new, not era, but in a way, we are wise, intelligent women.” Ellen	
		«It has been very much up to me to figure things out myself, and I’m the type of person who never gives up if there’s something I think I need to fix; I just keep going. But it’s very exhausting when you are really at the breaking point yourself» Anne	
**3.3**	**Menopause as an awareness of a valuable transition**	“I feel, it’s a bit like a ‘reset.’ In a way, it involves focusing more on self-care, and again, it relates to empty nest syndrome and all of that, but you are somewhat urged to take yourself seriously, simply put, to be kind to yourself. I think that in some way, you’ll come out stronger if you stick to that.” Anne	
		“That’s what I need to celebrate. I have entered a transition to becoming a mature woman, right? It’s about saying, ‘Oh, it actually feels good,’ if you can embrace that. So, I’m working on embracing the mature woman.” Ellen	

*SOC; sense of coherence.

#### 1. A transition from the unknown to normalisation – increasing comprehensibility

##### 1.1. The unknown takes over until the hot flashes bring clarity

All women expressed a challenge in comprehending what was happening in their bodies, not understanding that their psychological and cognitive symptoms could be related to hormonal changes. Even when they went to their GP for help, some felt that they did not gain clarity regarding their symptoms.

“The challenges I’ve had, what can I pin them on? That’s the problem, you know, when we are forgetful, right? And I’ve actually asked if I could get a dementia test, you know. Then it might turn out later that it’s stress. Is it menopause. Is it… I don’t know.” Louise

The understanding of being in menopause was strongly connected to hot flushes.

“You might not understand. It sneaks up on you, you know. But like, not all the symptoms are exactly... I was waiting for the hot flushes.” Anne

##### 1.2. Normalisation – it’s not only me

The need for menopause to be normalised was voiced by most participants. Many noted that the menopause is currently put on the agenda more frequently in society and found this to be helpful. Normalisation contributed to a broader comprehension of what the menopause encompassed and led to a sense of community with other women and a feeling of not being alone in this period.

“Yes, because I want to know about different experiences. ‘What I feel, do you feel the same? Is it like this for you? Do you experience it that way?' There is something about not being alone, that there are others who experience the same. It is normal to experience this low mood. It is normal to feel this way. That is what I want to confirm.” Sara

#### 2. From distress and silence to empowerment – managing menopause

##### 2.1. Why has no one told me how to face this phase of life?

Lack of knowledge about menopause, together with the silence that had surrounded it throughout their lives, left many women feeling frustrated. This knowledge gap made it difficult to know how to help themselves when struggling. Getting knowledge gave a feeling of manageability and safety, through understanding the symptoms, gaining acceptance and tools to manage challenges.

“Knowledge and security, really, go hand in hand for me.” Anne

Many actively sought out the dialogue around menopause to acquire new knowledge and gain insights from others’ experiences. Knowledge sources mentioned were the internet, podcasts, books, social media, family, colleagues and friends. Several expressed a desire for their GP to provide a reliable source of information, as a link to a website. At the same time, several participants expressed a sense of responsibility to seek knowledge on their own, acknowledging the busy schedule of the GP.

“I also have a responsibility myself to get information. The GP is very busy and can’t inform everyone about this.” Sara

##### 2.2. Let’s talk about it, but not before I am ready, and not about all symptoms

At the same time several reflected on the fact that the conversation about menopause was challenged by timing and interest. Many recognised that having knowledge before entering menopause could be advantageous, wishing they were better prepared, and that the GP had put menopause on the agenda. However, several felt they would have been sceptical about receiving this knowledge until they felt ready themselves.

“It can also be a case of 'no, that's not me,' right? So, it's really about both parts, I think. It's not just that; I believe there are many GPs who can contribute. But there needs to be a recipient who is willing to engage.” Ellen

Discussions often took place late in the development of symptoms. Despite experiencing symptoms, many did not address them until they suspected a connection to menopause. This led to a delayed understanding and management of their situation.

The conversation among women also faced challenges. Some expressed that not all women were interested in discussing menopause prior to experiencing it themselves.

“I can see that younger friends are like 'talk to my hand.' They don’t want to hear about menopause at all.” – laughs “And then I say, 'I wish it had been a bit more prepared, are you sure…?' And these are people who work in healthcare and are openminded. No, it's not relevant for them; they find it incredibly boring when the rest of us talk about menopause. Because they’re not there themselves.” Anne

Some symptoms were harder to discuss. Hot flushes were relatively easy to mention, while urogenital symptoms were reserved for private conversations. Several expressed that being open with their partner improved the understanding and acceptance of their own issues, while also opening for discussions about his own midlife challenges.

In the workplace, menopause discussions were influenced by age and gender of colleagues. Not all women felt comfortable addressing the topic at work, and some selected a few colleagues they shared their experience with. One participant shared her experiences with her closest younger coworkers, both men and women to teach them about menopause. Another felt that younger colleagues stigmatised midlife women. Still, several had acquired knowledge about menopause from colleagues, both before entering menopause themselves and through shared experiences.

##### 2.3. My doctor is important but has no clue – how is that possible?

Most women contacted their GPs’ office for help when experiencing symptoms. Several not knowing whether symptoms were connected to menopause and wanted to understand what was going on. They expected the GP to help them, but some ended up with the feeling of not getting help.

“I think, this affects half the population, so I don't understand why this isn't straightforward at a GP's office.” Anne

Some were referred to a gynaecologist without the GP involving in the treatment. Several felt they were left to figure out by themselves what was going on, and some got help outside the public health system.

One woman mentioned she noticed a change among GPs, observing that doctors today are more attentive to menopausal issues. Still, several of the participants experienced or assumed that their GP did not know enough about menopause. While menopause being a life phase for all women, some expressed despair over the lack of knowledge among doctors to adequately recognise, treat, and respect women during this period. Lack of time, medical mistreatment, misdiagnosis, breach of trust and the feeling of not being taken seriously or heard was part of the experiences.

Most women still regarded their GP as a vital support in their lives. Some emphasised that the doctor’s familiarity with them prior to entering menopause made the GP particularly important during this time. One of the women expressed genuine gratitude for the help her GP had provided.

##### 2.4. I want my doctor to be curious

Several recognised the GP’s extensive role with time pressure and many tasks. At the same time, some wanted the doctor to be curious, believing this would be beneficial also when it came to time management to facilitate a deeper understanding of the changes they were experiencing and enhance the ability to provide them with appropriate support.

“Instead of me sitting around trying to search and figure everything out while being on sick leave for months, I believe that a little curiosity from the GP can prevent that.” Anne

There was also a wish for the GP to emphasise the importance of taking care of one’s health during this period of life to cope better in a busy life.

##### 2.5. I can choose to influence by putting myself on the agenda

Acceptance of the challenges posed by hormonal changes was important for most of the women, and they also found self-care to be a valuable management tool for maintaining well-being during menopause.

“I actually feel that now it’s even more important to take care of myself and not to fight against it. It is a part of the cycle, and now I am getting help to ease the discomfort. But in a way, it's about accepting what is happening with my body, not resisting it. Instead, I try to do everything I can to alleviate the discomfort and make things better.” Elisabeth

#### 3. Finding meaning in the changes menopause brings

##### 3.1. Menopausal symptoms as a sign of an unwanted aging process

Some women experienced the menopause transition as a challenging sign of aging. It affected their sense of worth, self-image and femininity, and how they perceived themselves in society, at work and in relation to others. The symptoms the hormone changes induced, gave a feeling of not recognising themselves any longer, seeing the changes as unwanted and negative.

“There have been physical changes that I dislike. I dislike that my skin changes, that the smells change, and that I look different. It's all part of aging, right?” Dora

##### 3.2. This is important – I need to understand

The need to understand and sort out ‘what was what’ in the symptoms experiences was a motivation to manage and help themselves and increased the engagement and sense of purpose in navigating the challenges posed by menopause.

“I want to support the idea of approaching this with curiosity and a bit like, 'Okay, now we’re stepping into a new, not era, but in a way, we are wise, intelligent women.'” Ellen

Because of and despite bothersome symptoms, the women kept on seeking meaning, help and answers, with several visits to the GP and some to a gynaecologist. Some found support from other women or found information on their own, that helped them gain understanding. This understanding, in turn, enhanced their ability to cope.

“It has been very much up to me to figure things out myself, and I'm the type of person who never gives up if there’s something I think I need to fix; I just keep going. But it's very exhausting when you are really at the breaking point yourself.” Anne

##### 3.3 Menopause as an awareness of a valuable transition

Several of the women expressed new positive opportunities in the menopause transition. Changes brought about by menopause represented a shift towards improved self-awareness and self-care.

“I feel, it's a bit like a 'reset.' In a way, it involves focusing more on self-care, and again, it relates to empty nest syndrome and all of that, but you are somewhat urged to take yourself seriously, simply put, to be kind to yourself. I think that in some way, you'll come out stronger if you stick to that.” Anne

They recognised possibilities in a future without menstruation, and some appreciated that this phase of life reduced their sense of being objectified. They acknowledged their value in midlife, with experiences and opinions that they relied on. Several expressed a sense of freedom in no longer caring about what others thought about them. The aging process was also viewed positively by some, as it offered security, and gratitude.

“That's what I need to celebrate.” (Smiling) “I have entered a transition to becoming a mature woman, right? It's about saying, 'Oh, it actually feels good,' if you can embrace that. So, I'm working on embracing the mature woman.” Ellen

## Discussion

### Summary

Normalisation of menopause through knowledge increased all elements in Antonovsky’s Sense of Coherence. With knowledge, their comprehension grew and empowered management of this life phase. The GP was important to many of the women, though several experienced that the GP did not help them in this life phase. They acknowledged the busy schedule of GPs but wanted the GP to provide a reliable information source and expected the GP to have sufficient knowledge about menopause care. Even if menopause was associated with an unwanted aging process, they found meaning in this transition through increased focus on self-care and a growing sense of gratitude and self-worth.

### Discussion of results

Previous studies describe a knowledge gap both among women, GPs, and in the health care system.^10,12,14,23,24^ Our participants’ lack of knowledge influenced all elements of Sense of Coherence, and the elements were intertwined. Previous studies also show that it took time before women understood that the menopause had started^12,14,38^ and that health personnel did not meet their challenges.^12,14,39,40^ The *meaningfulness* component of *Sense of Coherence* enhanced the participants to seek understanding and empowered them to manage their experiences. Other studies show that concerns about symptoms and the feeling of not being seen motivate women to persist trying to understand and get help.^12,41^

One coping strategy was to normalise menopause through discussion with other women, but this conversation was difficult before they understood what was happening. Sergeant et al. noted that women struggled to find a language to discuss menopause as it was kept hidden in society, limiting normalisation through dialogue.^38^ The youth ideal of society can make women feel that they are expected to look and perform the same regardless of age.^12,38^ Studies have noted that this made menopause embarrassing, not something women wanted to be identified with.^18,42^ The growing attention around menopause in society was seen by all participants as positive, also showed in a Swedish study.^42^ The attitude to menopause is changing, both among women, in workplaces and educational systems.^43^ Several participants denied being embarrassed, they actively sought out discussions with other women to gain valuable knowledge. The community with other women helped, contributing to a better *Sense of Coherence.* This transition from the unknown to normalisation made the women feel less alone and helped them in “moving from uncertainty toward acceptance” as expressed in a study from Bahri et al.^44^

The experience of menopause is influenced by many factors; social context, cultural beliefs, expectations, attitudes and values around ageing and reproduction.^45^ The communication women engage in and the information received influence their perceptions on menopause.^9^ Normalisation of menopause in society may contribute to the understanding that this phase of life give diverse experiences, both in symptoms, and duration.^45^ Normalisation gives room for better conversations and challenges the negative narrative of the aging, sweaty menopausal woman, that associates this natural life phase with shame, aging and discomfort.^12,44^ At the same time, women express concerns for medicalisation with hormonal therapy as the ‘only‘ solution in TV programs that aim to educate on menopause.^14^ A Lancet editorial (2024) stated that when MHT is promoted as ‘a panacea’ it narrows conversations about this midlife phase, hides the need to enable informed, individualised decisions on optimal management, and may lead to overmedicalisation.^46^

Increased knowledge results in a greater demand for help from GPs concerning menopause care.^25,47^ Women express frustration about doctors’ lack of knowledge, especially since menopause is a “key part of half the population’s lives” as expressed by a participant in a study by Munn et al.^13^ In our study, several were surprised as they expected GPs to have knowledge about this natural life phase. Doctors do not always feel equipped to help women in menopause, lacking education on the topic.^23,24^ Educational shortcomings on menopause can lead to therapeutic inertia, misinformation and limited engagement from health personnel.^48^

Health providers’ lack of time and knowledge about menopause has been highlighted as a reason for difference in menopause care given by GPs,^23,25^ while it is also argued that the integration of psycho-socio-cultural factors into doctors’ understanding of menopause, could help effectiveness of menopausal care.^38^ This is in line with the concept of “time needed to treat”.^49^ As highlighted in a Maturitas editorial (2025): “Repositioning midlife hormonal health as central to healthy ageing must become a collective priority across disciplines and healthcare institutions.», and that inadequate training of health personnel might lead to poor help and erode trust in the provider-patient relationship.^48^

Several participants expressed that their regular GP was important to them in this phase of life due to the already established patient-doctor-relationship, a feature of the Norwegian GP system shown to improve health.^50^ The menopause transition collides with other challenges in women’s lives, as caring for children and parents, increased demands in working life, financial and health concerns.^11,51^ This “midlife collision”^52^ might contribute to the feeling of not understanding, as the symptoms associated with a hectic lifestyle and menopause can exacerbate one another, and stress related symptoms might even be mistaken for being menopausal.^6^ With an established doctor-patient relationship, the GP is well placed to help clarify her symptoms and treatment options, but this requires the doctor to be up to date with current knowledge.

The participants did not expect the GP to give lectures on menopause, but rather to provide them with a reliable information source, as a website. There is need for trustworthy information sources, as information given in society might be confusing.^14,17,43^ Information needs to be easily accessible and validated and could be framed as public health campaigns.^13,42,43^ Balanced evidence-based information, both in society and clinical setting, about the changes in menopause can empower women to manage this life stage^45^ and could potentially reduce visits to primary care.^53^

The participants’ stories reflect a broad attitude to menopause, from struggling to gaining empowerment, with fluctuating Sense of Coherence and both enhanced and challenged SOC-components. Different attitudes were present within the same woman in the same interview, also known from studies done by Hvas.^54,55^ In a recent systematic qualitative review study, women reported a range of feelings and attitudes to menopause, both empowerment and liberation as well as vulnerability and loss; women saw menopause as a normal, inevitable part of aging and a possible empowering chapter of life with room for growth and self-discovery, but also a turbulent time being intertwined with other midlife challenges.^51^ Much of this was also expressed by the women in our study and by others; menopause make women review their lives, and this shifts their focus to their own development, in a positive process,^38^ like a new rhythm to adjust to, not something to fear or resist.^56^

## Strengths and Limitations

As menopause can be a sensitive topic, in-depth individual interviews were useful to gain insights into strategies that support women during this life phase. Using salutogenesis helped us develop a more generalised analysis then just describing the data.^35^

By recruiting participants through hairdressers, we wanted to reach women with diverse reflections on menopause, regardless of whether they consulted their GP or used the internet and social media, and to ensure inclusion of women from both rural and urban areas. We managed to include women from various socioeconomic classes. All women expressed gratitude for taking part in this study, suggesting they did not feel pressured to participate through their hairdresser.

This recruitment method has limitations, introducing selection bias, as not all women attend hairdressers or talk to their hairdresser about menopause. The results are limited due to the homogeneity of participants, all of whom were cisgender, white, ethnically Norwegian women experiencing natural menopause. The hairdressers decided who to ask, and convenience sampling was part of their strategy, this giving potential for gatekeeping bias.

Menopausal experiences are shaped by many factors. We agree that further studies are needed to include women with diverse ethnicities and religion, gender-diverse individuals and women with different onset of menopause.^25,57^

## Conclusions

Gaining knowledge about menopause was an essential coping strategy for menopausal women to understand the symptoms, and to manage and give value to this transition. Women found their GP to be important and expected support from their GP during this phase, but several did not get the help they needed. Women value the increased attention around menopause in society, giving a feeling of normalisation and not being alone. Still, they express the need for reliable information about menopause from health professionals and government. This study highlights the importance of informing women, the public, and doctors, to improve menopause awareness, understanding and management, to enhance midlife women’s quality of life.

## Supplemental material

Supplemental material - Finding sense of coherence in the menopause transition - A qualitative interview study with menopausal women in Norway Supplemental material for Finding sense of coherence in the menopause transition - A qualitative interview study with menopausal women in Norway by Marianne Natvik, Mette Brekke, Siri Vangen, and Holgeir Skjeie in Women’s Health.

## Data Availability

To ensure participants confidentiality, the supporting research data are not publicly available. Data can be made available upon reasonable request to first author (MN). The raw data set is in Norwegian only.*
