# Supplementary material for: Finding sense of coherence in the menopause transition - A qualitative interview study with menopausal women in Norway
Source: Womens Health (Lond). 2026 Jun 10;22:17455057261459816. doi: 10.1177/17455057261459816 (PMC13254421; doi:10.1177/17455057261459816)
Supplement: Supplemental material - Finding sense of coherence in the menopause transition - A qualitative interview study with menopausal women in Norway [file sj-pdf-1-whe-10.1177_17455057261459816.pdf]

**PROJECT: Information needs regarding menopause**
**– women's needs and the role of general practitioners**

**Last version INTERVIEW GUIDE for sub-study 2**

| | |
|---|---|
| Sub-study 2 | Coping strategies and knowledge needs among women in the menopause transition |
| Method | Individual interviews with Norwegian women who are in the menopause transition |
| Interviewer | Marianne Natvik, Ph.D. candidate, Department of General Practice, University of Oslo |

**The intention of this interview guide**
The purpose of the interview is to create a good dialogue with the woman, so that I can understand her experiences and perspectives.
The interview guide is a guideline for what I want to include in the interview. My role as the interviewer is to help create a safe atmosphere and meaningful conversation. This is more important than the order of the questions, or that all questions are asked. However, the guide should support maintaining focus in the conversation.

**0. INTRODUCTION**

- Welcome and oral and written consent
  Thank her for participating in the conversation – I value her time
  Emphasize voluntary participation, she can withdraw whenever she wants
  Ensure consent
- Introduce myself, my interest in menopause.
  I am not a clinician in this setting.
  If she has any general medical questions she is curious about, we can discuss that after the interview.
- Introduce the project
  Our research question is about exploring coping strategies, information needs, and the role of general practitioners for women in menopause.
- The woman's role
  In this context, I am interested in her story and experiences.
- No answer is wrong. There is no definitive answer to any question.
- She can contribute new knowledge that can help others.

**1. Can you tell me a bit about yourself?**
Include: age, country of birth, where she lives in the country, education, profession, marital status, and the age and gender of her general practitioner.

**2. What are your thoughts about participating in this study we are conducting here?**
Why have you chosen to join?
This question is asked to elicit the woman's attitudes toward menopause.

**3. What are your thoughts on menopause as a period in a woman's life?**

**4. Can you tell me about your journey into menopause?**
Include: what symptoms
How it has potentially affected daily life.

How do you feel about the changes related to menopause?

How have you dealt with the symptoms?
Has anything helped you during this time? How? Do you have thoughts on why?

Has anything not worked? Why?
What do you do to help/support yourself?

Work: Has menopause affected your work situation? How and why?
Relationships: Has menopause affected your relationships? Which ones, how, why?

Has menopause affected your view of yourself and life? How and why?

**6. What significance has information about menopause had for you?**
Why/why not?
Perhaps from where? Was it useful?
When did you seek information?
Is there something you wish you had known earlier that you now know? Perhaps when?
Who or what could have informed you? Why would that have been desirable?
Has information affected

- Your understanding of menopause?
- Your attitude towards menopause?
- The dialogue with others?
- Your ability to get help?

**7. What role has your general practitioner had?**
If she had contact with the GP:
How and why have you been in contact with your general practitioner during this period?
If the general practitioner should help during this time, do you have any thoughts on when, how, and in what situations?

If no contact:
Why not?

**8. Is there anything you would like to say in conclusion that hasn't come up earlier in the conversation?**

**9. How did you find participating in this?**
Answers here can be used to adjust future interviews.
